# College and Hospital Notes

**Published:** 1908-03

**Authors:** 


### COLLEGE AND HOSPITAL NOTES.

Examination for internes to the House Staff of the City Hospital, New York, will be held on March 27, and 28, 1908 in New York City. The City Hospital has a large general service with about 800 beds, comprising all branches of medicine, and the length of service is 18 months. All applications for the position should be addressed to the Chairman of the Examination Committee, Dr. Smith Ely Jelliffe, 64 West 56th St., New York.

---

The Buffalo General Hospital will construct an annex on Goodrich street, just across the way from the main hospital building at an estimated cost of $35,000. A permit has been issued by the bureau of building and construction will begin in the spring. The building will be two stories high and fireproof.

The plans and expense are part of those arranged for under the will of the late Dr. D. W. Harrington of Cold Spring, who gave a large amount of money to the hospital.

---

The German Deaconess's Hospital having been reorganised, the board of directors has determined upon the establishment of a training school for nurses. This would seem to be a wise policy and it should receive the indorsement of all staunch friends of the hospital.

---

The Lockport City Hospital has been formally turned over by the contractor to the city authorities. The building cost $15,000, of which amount the city gave one third, Augustus Keep one-third, and the Hospital Aid Association raised the remaining third. The rooms in the building have been furnished by different persons, Mrs. John Hodge and Mrs. A. S. Beverly furnishing two of the larger rooms on the second floor. The Masons furnish the largest ward in the building. The operating room will be furnished by the Morrison Evans estate.

---

THE village of Medina is projecting a hospital and plans are beginning to take shape. An association is being incorporated which includes the names of 100 prominent citizens. Several sites are under consideration and a sum of money amounting to $300.00 left over from old home week now in the hands of the business men's association, is expected to become available as the nucleus of a building fund.

---

THE Buffalo Homeopathic Hospital realised a snug sum from the special matinee of the Bohemian Girl given February 12, 1908, by the Stewart Opera Company. The money is placed to the credit of the fund for the nurses' training school.

---

THE University of Buffalo observed University day, February 22, 1908, by the usual public exercises at the Teck theater. An address on Washington was given by Mr. Carleton Sprague, of Buffalo. Music was furnished by the U. of B. glee club and the 65th regiment band. The faculty luncheon was served at Miss Vincent's tea room.

